# Thanksgiving reflections

**DOI:** 10.1051/ject/2025068

**Published:** 2025-12-17

**Authors:** Raymond K Wong

As Thanksgiving approaches here in the United States, I find myself reflecting on gratitude – and there is so much to be thankful for within the JECT community. At the top of that list are our incredible peer reviewers.

Each year in our final issue, we recognize all those who have contributed at least one review over the past 12 months. While compiling this year’s list, I was reminded again of how fortunate we are to have such a diverse and dedicated group of professionals volunteering their time and expertise.

Our mission is to serve readers and, ultimately, the patients under their care. But the heart of our work lies with our authors – the contributors who trust us with their research. For them, the peer review process is essential, and the quality of those reviews shapes their experience with the journal. Thoughtful, constructive feedback not only improves manuscripts but also encourages authors to return and share more of their work with us.

Peer review is a confidential process, so many readers may not realize the effort involved. As editors, we see firsthand how detailed, creative, and generous these reviews often are. Even when a manuscript receives a “Reject, Rewrite, and Resubmit” decision, authors are rarely discouraged because they leave with clear guidance – and many resubmissions ultimately succeed.

I am especially grateful for the breadth of expertise among our reviewers, including specialists beyond perfusion. Their insights strengthen the journal immeasurably. I also appreciate how experienced reviewers help mentor newer ones, ensuring that our high standards endure. If you are a previously published perfusionist – or simply passionate about advancing our field – I encourage you to reach out to us and join this effort.

This season, on behalf of the Associate Editor team and myself, I want to say a heartfelt **thank you** to every reviewer who has given their time and talent to JECT. Your work makes a difference – not just for authors, but for readers and patients everywhere.

Warm wishes for a joyful Thanksgiving. We are truly grateful to you.

Associate Editor Team: Laura Dell’Aiera, Erick McNair, Luc Puis, Patrick Weerwind

